# Ecological Processes Shaping Microbiomes of Extremely Low Birthweight Infants

**DOI:** 10.3389/fmicb.2022.812136

**Published:** 2022-02-28

**Authors:** Christos Zioutis, David Seki, Franziska Bauchinger, Craig Herbold, Angelika Berger, Lukas Wisgrill, David Berry

**Affiliations:** ^1^Division of Microbial Ecology, Department of Microbiology and Ecosystem Science, Centre for Microbiology and Environmental Systems Science, University of Vienna, Vienna, Austria; ^2^Division of Neonatology, Department of Pediatrics and Adolescent Medicine, Pediatric Intensive Care and Neuropediatrics, Comprehensive Center for Pediatrics, Medical University of Vienna, Vienna, Austria; ^3^Joint Microbiome Facility of the Medical University of Vienna, University of Vienna, Vienna, Austria

**Keywords:** neonatal microbiome, ecological processes, community states, Markov model, microbial community assembly

## Abstract

The human microbiome has been implicated in affecting health outcomes in premature infants, but the ecological processes governing early life microbiome assembly remain poorly understood. Here, we investigated microbial community assembly and dynamics in extremely low birth weight infants (ELBWI) over the first 2 weeks of life. We profiled the gut, oral cavity and skin microbiomes over time using 16S rRNA gene amplicon sequencing and evaluated the ecological forces shaping these microbiomes. Though microbiomes at all three body sites were characterized by compositional instability over time and had low body-site specificity (PERMANOVA, *r*^2^ = 0.09, *p* = 0.001), they could nonetheless be clustered into four discrete community states. Despite the volatility of these communities, deterministic assembly processes were detectable in this period of initial microbial colonization. To further explore these deterministic dynamics, we developed a probabilistic approach in which we modeled microbiome state transitions in each ELBWI as a Markov process, or a “memoryless” shift, from one community state to another. This analysis revealed that microbiomes from different body sites had distinctive dynamics as well as characteristic equilibrium frequencies. Time-resolved microbiome sampling of premature infants may help to refine and inform clinical practices. Additionally, this work provides an analysis framework for microbial community dynamics based on Markov modeling that can facilitate new insights, not only into neonatal microbiomes but also other human-associated or environmental microbiomes.

## Background

Preterm birth remains one of the major risk factors for acute as well as long-term adverse health outcomes. Even in high- and middle-income countries, premature birth has been estimated to account for over 50% of neonatal deaths (Blencowe et al., 2013). Extremely low birthweight infants (ELBWI) are at high risk for suffering prematurity-related complications. Among the most critical complications are necrotizing enterocolitis (NEC) (Samuels et al., 2017), early- and late-onset sepsis (EOS and LOS, respectively) (Escobar et al., 2014; Afonso and Blot, 2017), bronchopulmonary dysplasia (BPD) (Thekkeveedu et al., 2017), retinopathy of prematurity (ROP) (Quimson, 2015) and intraventricular hemorrhage (IVH) (Poryo et al., 2018). These complications are not only an acute risk, but can lead to lasting neurodevelopmental impairment (Stoll et al., 2004). Microbial colonization plays a key role in the maturation and function of the immune system (Geva-Zatorsky et al., 2017). This is particularly important for extremely preterm infants, as they are frequently exposed to invasive procedures such as catheterization, intubation and assisted ventilation, which are potent sources of nosocomial infections during intensive care (Ramasethu, 2017).

Extremely low birth weight infants, which harbor lower-diversity microbial communities compared to neonates with higher birth weight (Chernikova et al., 2018), are particularly prone to infections due to their naive immune system, less effective mucosal and epithelial barriers (Weström et al., 2020), diminished complement components, and impaired function of antigen-presenting cells (Sadeghi et al., 2007; Schüller et al., 2013; Wisgrill et al., 2016; Jong et al., 2017). Early gut colonization by pathogenic or immunomodulatory bacteria can distort the fragile immune homeostasis in the premature gut and predispose neonates to disease (Mazmanian et al., 2008; Wlodarska et al., 2015). For example, research suggests that NEC, one of the most severe complications for ELBWI, is preceded by alterations in the gut microbiome marked by decreased bacterial diversity and blooms of Gammaproteobacteria and bacilli (Morrow et al., 2013; Warner et al., 2016). Studies on the initial assembly of the human microbiota have been mostly focused on the gut community in term (Palmer et al., 2007; Koenig et al., 2011; Sharon et al., 2013; Hill et al., 2017) and preterm infants (Rosa et al., 2014; Ho et al., 2018). Recent efforts to describe spatiotemporal community dynamics within and across multiple body sites in premature infants have provided first insights into assembly patterns (Costello et al., 2013; Olm et al., 2017; Grier et al., 2018; Younge et al., 2018; Tirone et al., 2019), but the ecological processes underlying initial community assembly remain poorly understood. Generally, community dynamics can be described by four fundamental ecological processes: (1) selection, the fitness difference between species, (2) drift, stochastic changes in species abundance, (3) dispersal, the ability of species for movement across space to new sites, and (4) speciation (Vellend, 2010). Essentially, community assembly can be influenced by both deterministic and stochastic processes to a degree that is dependent upon changes of environmental factors over time and space (Stegen et al., 2012). In this study, we monitored amplicon sequence variant (ASV) relative abundances on the skin, gut and oral cavity over the first 2 weeks of life in 15 ELBWI in order to better understand the process of *de novo* assembly of bacterial communities and to determine the relationship between communities at different body sites. We characterized bacterial diversity and inferred the ecological processes governing community assembly. Additionally, we evaluated associations between microbiome data and clinically relevant parameters such as gestational age, delivery mode, inflammatory response, and disease diagnosis. We then developed an analysis framework that identifies discrete community structures and interprets the observed temporal microbiome trajectories as a Markov process of transitions between community states.

## Materials and Methods

### Study Cohort and Sampling

We recruited a cohort of 15 ELBWI (defined as having a birth weight < 1000 g) hospitalized in the neonatal intensive care unit (NICU) at the General Hospital of Vienna/Medical University of Vienna. Exclusion criteria were chromosomal aberrations, congenital malformations, inherent metabolic disorders, and maternal chronic infections. Due to the high risk of necrotizing enterocolitis in this high-risk patient cohort, all infants received pasteurized human donor milk or their own mother’s milk in the first few weeks of life. Infant nutrition was supplemented with parenteral nutrition during the study period (Supplementary Figure 1) and combinations of broad-spectrum antibiotics (Supplementary Figure 2) were administered prophylactically during the study period. Patients were sampled at four time points over the first 2 weeks of life (postnatal days 1, 3–4, 7–8, and 14–16). Stool was collected and chest skin and oral cavity were sampled using ESwab™ (COPAN diagnostics) swabs. All samples were immediately stored at −80°C. The study was approved by the ethics committee of the Medical University of Vienna (EK No. 1175/2016).

### Clinical Definitions

Bronchopulmonary dysplasia (BPD) was defined as supplemental oxygen treatment or oxygen plus respiratory support at 36 weeks postmenstrual age (Higgins et al., 2018). Retinopathy of prematurity (ROP) was diagnosed and staged according to the international consensus guidelines (International Committee for the Classification of Retinopathy of Prematurity, 2005). The severity of intraventricular hemorrhage (IVH) was defined as grades 1 to 4 according to the modified Papile classification (Papile et al., 1978). Statistical analyses relating ROP and IVH to microbiome data were performed without considering disease severity due to limited sample size of some disease grades.

Elevated IL-6 was defined as IL-6 > 150 pg/ml. Early-onset clinically suspected inflammation (CSI) was defined as IL-6 > 150pg/ml on day 1 or 2 of life, but with negative blood culture results (i.e., elevated inflammatory markers, but no clinical diagnosis of sepsis). Late-onset sepsis (LOS) was defined according to the NEO-KISS protocol (Gastmeier et al., 2004) for nosocomial infection surveillance for preterm infants. Clinical LOS as well as LOS with coagulase negative staphylococcus (CoNS) were defined as an episode with the following characteristics: > 72 h of life, empiric antibiotic therapy ≥ 5 days, no apparent infection at another body site, and additionally fulfilling any two of the following criteria: temperature > 38°C or < 36.5°C, temperature instability, tachycardia, bradycardia, apnea, hypotension, hyperglycemia, metabolic acidosis, prolonged recapillarization time or positive blood infection parameter (C-reactive protein > 2 mg/dl or IL-6 > 50 pg/ml). Culture-positive LOS was defined as a clinical infection as described above with the additional growth of a pathogen in the corresponding blood culture. Oxygen supplementation (DOS) was defined as the cumulative number of days with at least 12 h/day of FiO_2_ > 21% up to the day of sampling. Days of mechanical ventilation (DMV) was defined as the number of days with mechanical ventilation support up to the day of sampling. Antibiotic administration was measured as a cumulative index of days of antibiotic given accounting for parallel antibiotics prescription (antibiotic prescriptions per day multiplied by the number of days of administration).

### 16S rRNA Gene Amplicon Sequencing

For nucleic acids extraction, we used 100 mg of stool or 500 ul swab solution for skin and oral samples, after vigorous vortexing to release cells from the swab. Nucleic acids were extracted using a phenol-chloroform bead beating protocol (Griffiths et al., 2000). Barcoded amplicon libraries were prepared according to a two-step PCR protocol as described previously (Herbold et al., 2015). Briefly, the extracted DNA was PCR amplified for 30 cycles in total, using a universal primer pair S-D-Bact-0341-b-S-17 [5′-CCTACGGGNGGCWGCAG-3′] and S-D-Bact-0785-a-A-21 [5′-GACTACHVGGGTATCTAATCC-3′] that targets the V3-V4 hypervariable regions of the 16S rRNA gene. Amplicon libraries were purified and normalized in equal molar quantities with the *SequalPrep*™ Normalization Plate Kit (Invitrogen, United States) before pooling. The preparation was performed on an automated liquid handling workstation (Beckman Coulter, United States). Sequencing on the Illumina MiSeq platform (2 × 300bp) was performed at Microsynth AG (Balgach, Switzerland).

### Sequence Data Pre-processing

Reads were demultiplexed using an in-house python script. A custom pipeline built on *Qiime2* (Bolyen et al., 2019) was developed for processing the sequence data. Specifically, reads were processed into amplicon sequence variants (ASVs) using the Divisive Amplicon Denoising Algorithm (*DADA2*) (Callahan et al., 2016) without the pooling option. We extracted sequences from the *SILVA* database (SILVA 132, 99% OTUs) and trained a classifier (bayesian module from *Qiime2*) specific to the amplified region for taxonomic assignment of ASVs (Bokulich et al., 2018). Due to the very low read yield of the negative control libraries, no reads passed the DADA2 pipeline. Thus, we examined libraries for potential contaminants by taxonomically classifying the raw reads from negative control samples by best BLAST hit to the NCBI 16S rRNA database. Genera with > 10 reads were considered contaminants (and accounted for on average ∼0.8% of total sequences in the libraries from the patient sample). We then removed any genus that was highly correlated across the dataset with the contaminant genera (Pearson r > 0.9), as well as previously described PCR reagent contaminants (∼11% of total sequences) (Salter et al., 2014). Additionally, we removed ASVs that were detected in higher abundance in samples from other datasets in the same sequencing run as potential cross-contamination (∼0.13% of total sequences). Libraries were rarefied to 600 reads per sample for subsequent analysis after evaluating rarefaction curves to ensure inclusion of the maximum number of samples given no richness underestimation (Good’s coverage min = 0.995, max = 1, CI_95_ = [0.9986984, 0.99918778]; Supplementary Figure 3).

### Ecological Statistical Analyses

We measured alpha diversity (Shannon index, evenness, and richness) with the skbio.diversity.alpha_diversity function from *scikit-bio* python module and Beta diversity (*Bray-Curtis* dissimilarities) with the scipy.spatial.distance.pdist function from *scipy* python module (Virtanen et al., 2020). Differential abundance analysis was carried out with the *MetagenomeSeq* R package (Paulson et al., 2013). For multivariate analysis, we used the *adonis* (PERMANOVA, using 1,000 permutations) and *envfit* functions from the *vegan* R package, with permutations constrained to the patient samples (Dixon, 2003). The significance between groups was assessed with ANOVA or the Wilcoxon rank-sum test with the *aov* and c*ompare_means* functions in R. We applied k-means clustering to partition samples based on their *Bray-Curtis* dissimilarities. We then evaluated clustering efficiency by comparing silhouette scores for individual clusters to the average Silhouette score (>0.67) as well as maximization of the Calinski harabasz criterion (Supplementary Figure 4). We constructed co-occurrence networks from pairwise spearman correlations that were calculated from ASV abundances across samples. Only ASVs present in at least 2 of the respective samples were used for the analysis. The significance of the calculated correlation coefficients was estimated by comparison to a null distribution. We obtained this null distribution by shuffling the ASV abundances across samples and re-calculating spearman correlations 1000 times. The resulting *p*-values were then corrected for multiple comparisons using the method of Benjamini and Hochberg (1995) and only observed correlation coefficients with a *p*-value < 0.01 were used for further analysis. Correlation coefficients were calculated using the function *cor* from the *stats* package in R. Network visualization was done with *Cytoscape* 3.8.0 (Shannon et al., 2003).

### Analysis of Ecological Processes

A maximum likelihood phylogenetic tree was reconstructed with *IQ-TREE* (Nguyen et al., 2015) based on the TIM3e + R5 DNA model, inferred with *ModelFinder* (Kalyaanamoorthy et al., 2017). Distance based RDA and phylogenetic diversity was calculated in R using the *vegan* package and *ordistep* function, as well as the *picante* package (Kembel et al., 2010). Null-model analysis was used to analyze the β-mean-nearest taxon distance (βMNTD) for all pairwise comparisons within each body site. The difference between observed βMNTD and the null model is given in units of standard deviation (of the null distribution) and termed βNTI (β-nearest taxon index). We interpret βNTI values according to previously established criteria (Stegen et al., 2013) as follows: scores greater than + 2 indicate variable selection pressures; scores near zero indicate dominance of stochastic processes; and scores less than −2 indicate homogeneous selective pressures. In a second step, the RCBray index was used to characterize stochastic processes. | RCBray| < 0.95 indicates that in a pairwise comparison, communities share as many species as expected by chance, indicating that drift acts alone. RCBray < −0.95 indicates homogenizing dispersal, as communities share more species than expected. RCBray > 0.95 indicates dispersal limitation, as fewer species than expected are shared.

### Estimation of Conditional Probabilities

For each infant, we estimated the conditional probability of detecting an ASV at a certain body site conditioned upon its detection at another body site, as follows:


$$P\,\left({\text{s}}_{1}|{\text{s}}_{2}\right)=\frac{P\,\left({\text{s}}_{2}|{\text{s}}_{1}\right)\,P\,\left({\text{s}}_{1}\right)}{P\,\left({\text{s}}_{2}\right)}$$

where, s_1_ is one body site and s_2_ is another. Similarly, we obtained the transition probability between community states for each body site by estimating the probability of a current community state, conditioning upon the community state at the previous time point.

### Markov Modeling

Markov models are stochastic models that assume that each future state depends solely on the current state. This assumption is reasonable for systems with frequent disturbances. We constructed a Markov chain from the previously estimated transition probabilities between community states and estimated their stationary frequencies. These are estimates of a future converging point in the process where the probability distributions will no longer change. Using these stationary or steady state frequencies, we can predict the equilibrium point of our transition model across community types. States in a Markov chain are categorized into transient and recurrent states. Recurrent states are those which are estimated to have a probability of one for returning to this state, whereas transient states do not. We used the functions *steadyStates* and *summary* from the *markovchain* package in R (Spedicato, 2017) in order to construct the Markov chain, estimate steady states, and explore their characteristics.

## Results

### Description of the Cohort

In our ELBWI cohort, the mean gestational age (GA) was 24.67 (± 1.12) weeks and the mean birth weight was 731 (± 116) g. Patient characteristics and the frequency of short-term morbidities are summarized in Table 1. The same probiotic supplementation (Antibiophilus^®^ - *Lactobacillus casei*) as well as parenteral and enteral feeding regimens were applied to all patients over the entire study period. Sampling was not synchronized across infants, although there were overlaps in hospitalization.

**TABLE 1:** Summary of cohort characteristics.

	Mean ± SD
Gestational age (weeks)	24.67 ± 1.12
Birth weight (g)	731 ± 116.19

	**N (%)**

**Gender**	
Female	8 (53)
Male	7 (47)
**Mode of delivery**	
Cesarean section	11 (73)
Vaginal birth	4 (27)
**IVH**	
No	10 (67)
Stage 1	4 (27)
Stage 2	1 (6)
**BPD**	
No	10 (67)
Yes	5 (33)
**ROP**	
No	4 (27)
Grade 2	8 (53)
Grade 3	3 (20)
**NEC**	
No	15 (100)
**LOS**	
No	8 (53)
Yes	7 (48)
**CSI**	
No	2 (13)
Yes	13 (87)

*IVH, intraventricular hemorrhage; BPD, bronchopulmonary dysplasia; ROP, retinopathy of prematurity; NEC, necrotizing enterocolitis; LOS, late onset sepsis; CSI, clinically suspected inflammation.*

### Diversity of the Extremely Low Birth Weight Infants Microbiome

**FIGURE 1:**
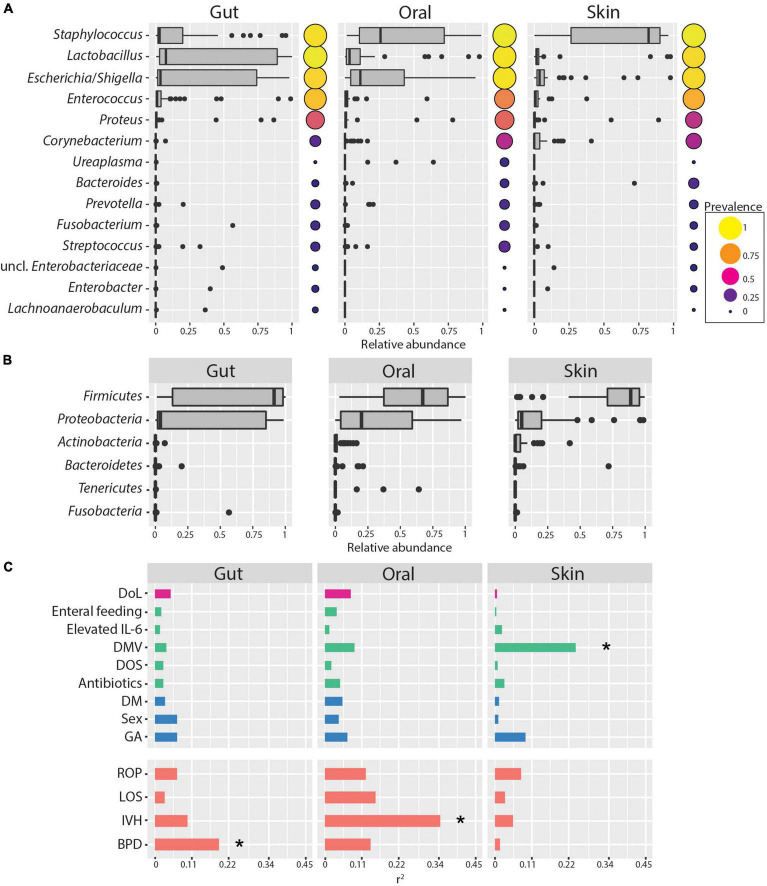
Skin, oral cavity, and gut microbiome composition of ELBWI. **(A)** Relative abundances of bacterial genera across body sites. Genera with >0.2 relative abundance in at least one sample are shown. Circle size and color indicates genus prevalence in the dataset. **(B)** Relative abundances of phyla across body sites. **(C)** Association of microbiome composition with clinical parameters and disease outcome (using the *envfit* function. Pearson correlation coefficient [r^2^] on *X*-axis). DoL, days of life; elevated IL-6 = IL-6 > 150 pg/ml in the time window of 2 days before to 2 days after sampling; DOS, number of days with at least 12 h per day of FiO_2_ > 21% up to the day of sampling; DMV, days with mechanical ventilation support till the day of sampling. Antibiotics = cumulative index for days of antibiotic administration accounting for different compounds per day; GA, gestational age; DM, delivery mode; ROP, retinopathy of prematurity; IVH, intraventricular hemorrhage; BPD, bronchopulmonary dysplasia. *p-value < 0.01.

Skin, oral cavity, and gut microbiome composition was obtained by amplicon sequencing of the V3-V4 regions of the 16S rRNA gene at four time points over the first 2 weeks after birth. In this period, ELBWI were colonized by low diversity microbial communities on all tested body sites. *Firmicutes* and *Proteobacteria* were the most abundant phyla across all body sites, predominantly due to the abundance of the genera *Staphylococcus*, *Escherichia/Shigella* and *Lactobacillus* (Figures 1A,B). *Staphylococcus* was on average four times more abundant (Cohen’s d _skin–gut_ = 1.5) in skin samples compared to gut samples and twice as abundant (Cohen’s d _skin–oral cavity_ = 0.64) compared to oral cavity samples, whereas *Lactobacillus* and *Escherichia/Shigella* were three times more abundant in gut communities compared to the skin (Cohen’s d _gut–skin_ = 0.67, Cohen’s d _gut–skin_ = 0.55, respectively). While *Escherichia*/*Shigella* was detected in similar abundance in gut and oral samples (Cohen’s d _gut–oral cavity_ = 0.08), *Lactobacillus* abundance remained half as low in oral samples (Cohen’s d _gut–oral cavity_ = 0.5) (*ANOVA* - *Staphylococcus*; *p* < 0.001, *Escherichia/Shigella*; *p* = 0.003, *Lactobacillus*; *p* = 0.027, respectively). However, similar communities were found at all body sites, and body site was only able to explain a minor, though statistically significant, fraction of the microbiome variation across the entire dataset (PERMANOVA: *r*^2^ = 0.09, *p* = 0.001). Clinical parameters such as delivery mode and gestational age at birth also explained little of the observed variability in the composition of the microbiome (Figure 1C), though the low number of patients delivered by spontaneous birth and the small range of gestational ages of study patients (24.67 ± 1.12) must be considered when interpreting these results. Strikingly, duration of mechanical ventilation was associated with changes in skin microbiome (*r*^2^ = 0.24, *p* = 0.007). Considering clinical diagnosis, BPD and IVH were significantly associated with gut and oral cavity microbiome composition, respectively (Figure 1C). However, we did not identify any ASVs consistently associated with disease diagnosis, suggesting that there were no robust ASV-level markers for disease in this cohort.

**FIGURE 2:**
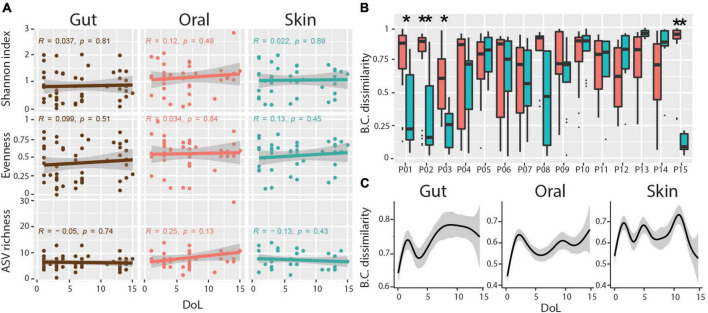
Diversity and body-site-specificity of microbiomes. **(A)** Alpha diversity metrics over patient days of life. Linear regression Pearson *r* and *p*-values are shown within each subplot. **(B)** Bray-Curtis (BC) dissimilarities of samples from the same body site compared to samples across body sites for each infant. Wilcoxon Rank-sum test, adjusted *p*-values * < 0.01, ** < 0.001. **(C)** Progression of Bray-Curtis (BC) dissimilarities (*Y*-axis) in ELBWI with increasing distance over time (*X*-axis). Smoothed lines result from locally estimated scatterplot smoothing (LOESS) and indicate trends of development.

Microbial community diversity remained low at all body sites over the first 2 weeks of life, with no significant trend for increasing ASV richness, evenness, or Shannon diversity during this period (Figure 2A). As previously mentioned, we observed a subtle association between microbial communities and body sites. However, only four of the 15 infants had significantly more similar communities within each body site compared to between body sites over the sampling period (Figure 2B). Additionally, we find no indication of community succession within this timespan, with no increase in Bray-Curtis distance of communities over time compared to the founder community within this period (Figure 2C). These data indicate that habitat filtering is largely overwhelmed by stochastic disturbance events.

### Ecological Processes Affecting Microbiome Assembly

**FIGURE 3:**
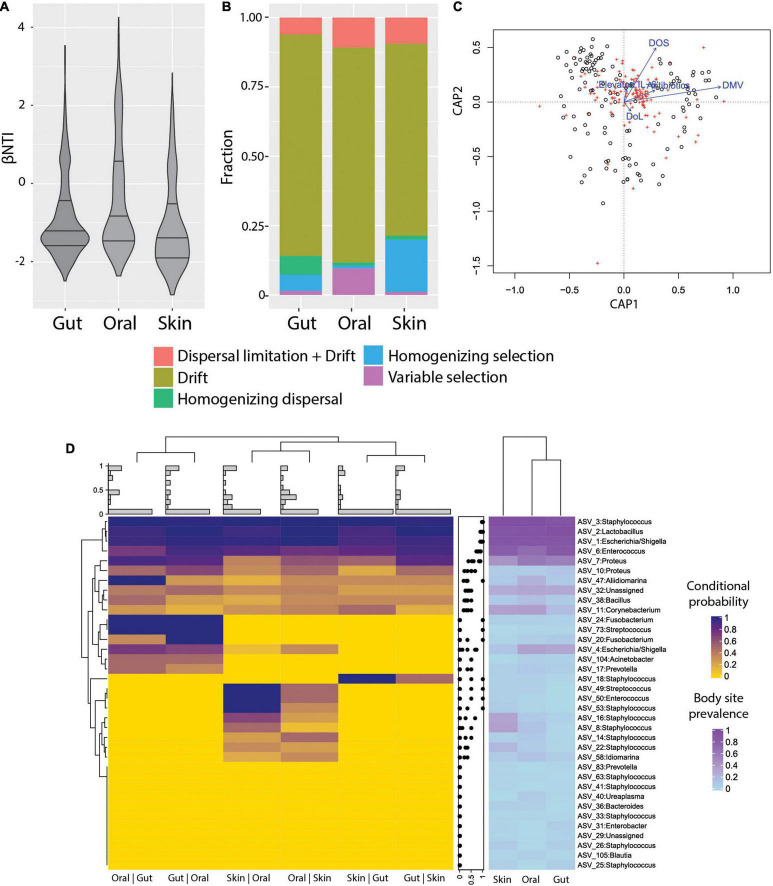
Ecological processes and microbiome assembly. (A) βNTI (beta Nearest Taxon Index) per body site. βNTI greater than + 2 standard deviations from the null-model indicate variable selection pressures; scores near zero indicate dominance of stochastic processes; and scores less than -2 standard deviations indicate homogeneous selective pressures. (B) Fraction of ecological processes predicted for each body site. (C) Canonical correlation analysis (CCA) plot, showing the relative proportion of βNTI variance explained by DMV, DOS, Elevated IL-6, Antibiotics, and DoL (proportion of constrained variance = 0.04). DoL, days of life; Elevated IL-6, IL-6 > 150 pg/ml in the time window of 2 days before to 2 days after sampling; DOS, number of days with at least 12 h per day of FiO_2_ > 21% up to the day of sampling; DMV, days with mechanical ventilation support till the day of sampling. Antibiotics = cumulative index for days of antibiotic administration accounting for different compounds per day. (D) Heatmap of conditional probability to detect an ASV at a body site when present at another (left). Distribution of values for each row (center). Heatmap for ASV prevalence in each body site (right).

To characterize the ecological drivers of microbiome assembly in different body sites, we compared the observed phylogenetic diversity to ecological null models and determined the contributions of selection, dispersal, drift, and dispersal limitation to community turnover in ELBWI using an approach established by Stegen et al. (2013) and (Table 2). We observed that stochastic processes (βNTI < | 2|) dominated community assembly across all body sites, but that deterministic processes were higher in the oral cavity than the other body sites (Figure 3A; ANOVA, *p* < 0.0001, Tukey *post hoc*: oral-gut and oral-skin *p* < 0.001). Analysis of the specific processes revealed that ecological drift dominated all body sites, but that there was elevated homogenizing dispersal in the gut (Chi-square test, *p* < 0.001), variable selection in the oral cavity (Chi-square test, *p* < 0.001), and homogenizing selection on the skin (Figure 3B; Chi-square test, *p* < 0.001). Canonical correlation analysis (CCA) revealed that mechanical ventilation, oxygen supplementation and antibiotic administration were significantly associated with βNTI values, though they only explained a small percentage of the observed variation (Figure 3C).

**TABLE 2:** Glossary of ecological terms.

Speciation	The creation of new species (Vellend, 2010)
Ecological drift	A stochastic element of the changes in the composition and diversity of species, due to their ecological equivalence (Vellend, 2010)
Environmental/habitat filtering/selection	Environmental factors which increase fitness of particular species (Vellend, 2010)
Dispersal limitation	The limitation of a species ability to spread/move across space, resulting in local communities that altogether define a metacommunity (Vellend, 2010)
Homogenizing dispersal	High levels of species dispersal which are able to homogenize community composition (Stegen et al., 2013)
Homogenizing selection	Strong selection that reduces compositional turnover for communities exposed consistently to the same environmental pressures (Stegen et al., 2015)

The detection of distinct community types that were not body-site-specific suggested limited dispersal limitation and/or environmental filtering of bacteria across body sites. In order to more deeply characterize the extent of habitat specificity of microbes and potential dispersal of microbes between body sites, we calculated the probability of detecting an ASV at a body site given that it is detected on another body site of the same patient (i.e., conditional probability). We found that very abundant ASVs are likely to be present in all sampled sites from the same patient, which is consistent with the neutral theory of community assembly (Volkov et al., 2003). However, the majority of ASVs were detected sporadically, and in some cases were exclusive to certain neonates (Supplementary Figure 5). Interestingly, ASVs were more likely to be detected in the gut if they were detected in the oral cavity (Posterior probability = 0.55), and vice-versa (Posterior probability = 0.56) compared to the other body site combinations. The oral cavity and skin had somewhat fewer ASVs with a high conditional probability of detection (Posterior probability = 0.42 and 0.37, respectively), and between skin and gut that was even lower (Posterior probability = 0.29 and *P* = 0.29, respectively) (Figure 3D). This suggests that either physical proximity between body sites or the relative similarity of environmental conditions (e.g., O_2_ levels, water content, or immune factors) could influence establishment of certain ASVs.

### Extremely Low Birth Weight Infants Microbiomes Have Distinct Community Structures

**FIGURE 4:**
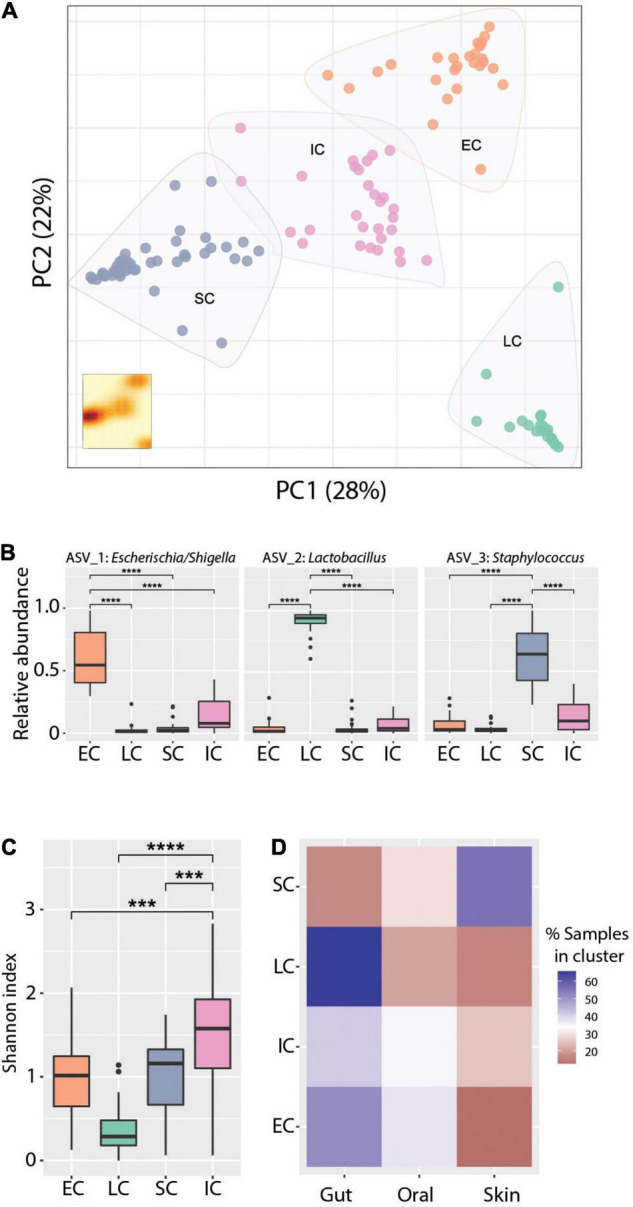
Cluster analysis reveals distinct community states. (A) Principal coordinates analysis ordination of Bray-Curtis dissimilarities. K-means clustering analysis indicates four community types based on Silhouette score - Calinski harabasz maximization. KDE plot (bottom right) displays sample density on the first two principal components. (B) Relative abundance of the dominant ASVs in the respective clusters, Wilcoxon Rank-sum test ****p-value < 0.0001. (C) Alpha diversity measured with the Shannon index across community types. Wilcoxon Rank-sum test ***p-value < 0.001, ****p-value < 0.0001. (D) Representation of community types (clusters) in the gut, skin, and oral cavity respectively, as percentage of the total number of samples assigned to each cluster. Blue and red color gradients indicate positive or negative deviation, respectively, from a uniform cluster distribution across body sites (i.e., 33%).

Principal coordinates analysis (PCoA) revealed clear clustering of samples (Figure 4A), though this clustering was not driven primarily by body site, age, or patient (Supplementary Figure 6). To further define and characterize these discrete community structures, we performed a cluster analysis optimization based on silhouette score maximization (Arumugam et al., 2011). This revealed four community structures (all clusters were well-supported, having an average silhouette score = 0.67), which were driven largely by differences in the relative abundance of the three most dominant ASVs in the dataset. Specifically, we identified three mono-dominated clusters (SC, EC, and LC), in which ASV_3: *Staphylococcus* [SC, mean = 0.61, *CI*_95%_ = (0.55,0.68)], ASV_1: *Escherichia/Shigella* [EC, mean = 0.6, *CI*_95%_ = (0.51,0.69)] and ASV_2: *Lactobacillus* [LC, mean = 0.91, *CI*_95%_ = (0.86,0.95)] were predominant, respectively (Figure 4B). We also observed a cluster of samples (IC) that had comparatively intermediate relative abundance for the above-mentioned ASVs, as well as a higher overall bacterial diversity (Figure 4C). The identified microbiome structures were unequally present at the studied body sites: we detected the LC cluster mainly in the gut, EC more frequently in the gut and less in the oral cavity, whereas SC was enriched in skin samples. Interestingly, the IC cluster appeared more evenly across all body sites (Figure 4D). The structural differences between those clusters were also reflected in their co-occurrence networks, which show distinct topologies and cluster-specific correlation patterns of ASVs (Supplementary Figure 7).

### A Probabilistic Framework to Characterize Microbiome Dynamics

**FIGURE 5:**
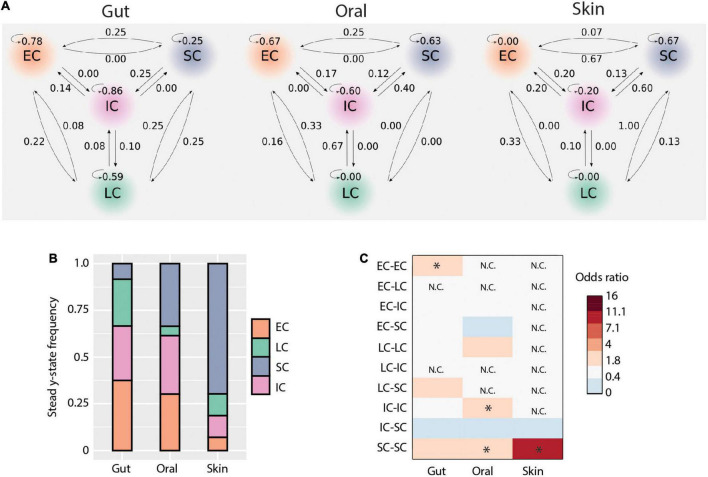
Microbial transmission and community dynamics. (A) Schematic representation of transition probabilities across body sites. (B) Community types steady-state frequencies across body sites. (C) Contribution of each community type comparisons to deterministic processes (i.e., bNTI values > | 2|). The heatmap shows the odds ratio of each comparison to contribute to observed deterministic processes for each body site. Significant values (i.e., when the lower bound of the 95% confidence interval was above 1) are indicated by an asterisk.

It has been suggested that initial microbial community assembly in low-birth-weight neonates is a stochastic process (Costello et al., 2013). As our data indicated that deterministic processes also play a role in assembly and that there are distinct microbiota compositional states, we next applied a probabilistic analysis framework to gain a deeper understanding of the observed dynamics. We modeled how the microbiome at each sample site changes over time as a Markov process, using conditional probabilities estimated from the data. Specifically, we estimated the probability of observing a certain state (community structure), conditioning on the state at the preceding time point, for each body site and each patient separately. This approach revealed new aspects of early-life microbiome dynamics (Figure 5A). In the gut, community structures were the most stable (mean prob. = 0.62) compared to the other body sites. Specifically, EC, IC, and LC were stable clusters (prob. > 0.5), while SC had equal probability to transition to other community structures. In contrast, SC was more stable in the oral cavity, and there was a higher net transition probability from IC to SC communities. LC appeared sporadically in the oral cavity and transitioned only to EC or IC. For the skin, we observed an overall lower community stability compared to other body sites (mean prob. = 0.21). However, there was a higher transition probability to the SC cluster from all other clusters. These results suggest that microbiomes at different body sites have different levels of community stability and characteristic transitions between community states.

In order to better understand the implications of these community state transitions, we next evaluated the long-term consequences of these dynamics by calculating their steady-state probabilities using Markov chain theory. Briefly, Markov chains are “memoryless” processes in which the transition from one discrete state to another depends solely upon the present state, but not the past. This assumption is reasonable for systems with frequent disturbances, such as would be expected considering the intensive clinical care practices administered to ELBWI. States can be classified into recurrent or transient, based on their presence or absence at steady-state (i.e., equilibrium). At all body sites, all clusters were found to be recurrent states. However, their steady-state frequencies differed across body sites (Figure 5B). EC and IC shared similar frequencies in the gut and oral microbiomes at steady state, while LC was higher in the gut and SC higher in the oral cavity. Notably, SC comprised ∼70% of the communities at steady state in the skin.

We then evaluated the contribution of each community type to the observed deterministic processes in each body site (Figures 3A,B). This revealed that in the gut EC clusters contributed significantly to deterministic assembly [Odds ratio: 3.3, 95% CI = (1.4,7.9)], whereas in the oral cavity IC and SC contributed to deterministic assembly [Odds ratio: 2.5, 95% CI = (1.1,6.0) and Odds ratio: 2.8, 95% CI = (1.3,6.4), respectively], and in the skin SC contributed to deterministic assembly [Odds ratio: 9.3, 95% CI = (1.3,69.2); Figure 5C]. These results are largely in line with the Markov modeling results, suggesting that community state stability is governed in part by deterministic ecological processes.

## Discussion

Previous studies of gut microbiome assembly in preterm and term infants have suggested that over the course of months to years there is a succession in microbial communities (Koenig et al., 2011; Rosa et al., 2014; Hill et al., 2017; Stewart et al., 2018). However, studies that have focused on early-life microbiome development at multiple body sites in preterm infants have concluded that there are stochastic dynamics and lack of body-site-specificity in the initial community assembly in the first 2 weeks of life (Costello et al., 2013; Olm et al., 2017). As extremely preterm infants are highly vulnerable to inflammation, infections and associated adverse outcomes for which the microbiome is known to play a role (Dobbler et al., 2017; Younge et al., 2019), we sought to develop an improved framework to better understand early microbiome assembly and to identify ecological factors driving this process.

In this study, we show that the initial colonization of the gut, oral cavity, and skin in ELBWI is dominated by *Firmicutes* and *Proteobacteria* in the first 2 weeks of life. This is reflected in the abundances of three predominant ASVs classified as *Escherichia/Shigella*, *Staphylococcus*, and *Lactobacillus* genera, respectively (Figure 1A). These taxa are commonly observed in human microbiome studies in neonates as well as adults (Schloissnig et al., 2013; Sharon et al., 2013). Over the first 2 weeks following birth, alpha diversity of microbiomes across all body sites was variable and did not have an increasing trend, suggesting limited enduring microbial succession in this time period (Figure 2A). Only four of the 15 infants had body-site specific microbiomes across the time course, indicating extensive colonization of similar microbes at different body sites for most patients. Interestingly, after evaluating Silhouette score maximization to define clusters, we found that microbiomes could be classified into four distinct community types that could be observed at all body sites, three of which were dominated by either *Escherichia/Shigella, Staphylococcus* or *Lactobacillus*, and a fourth which was more diverse and included all three of these prevalent genera at similar levels (Figure 4).

We observed that community assembly was driven largely by ecological drift, but that the oral cavity had elevated variable selection, which may be due to differences in oral immune factors among patients that impose different selection pressures. Homogenizing selection was highest for the skin, which may be due to the presence of *Staphylococcus* as a well-adapted skin bacterium. The gut had the lowest dispersal limitation, which may be because the gut has a higher number of bacterial cells compared to other body sites, which increases their dispersal probability. Our results suggest that deterministic assembly processes are detectable in the first 2 weeks of life, but that they are largely overwhelmed by stochastic processes. Indeed, chaotic microbiome dynamics may occur due to the many possible environmental disturbances encountered immediately after birth such as antibiotic administration, mechanical ventilation or nutrition (Aujoulat et al., 2014; Stewart et al., 2016, 2018). Despite the known beneficial role of breastfeeding in term infants (Bäckhed et al., 2015; Stewart et al., 2018), in part due to the enrichment of bifidobacteria (Makino et al., 2013; Lawson et al., 2020), we find that the amount of enteral feeding (mother or donor pasteurized milk) had no detectable effect on microbiome composition. This is in line with previous findings in preterm infants where nutritional exposures did not shape the microbiome within the first weeks (Gregory et al., 2016). The absence of bifidobacteria from our study could be attributed to the effect of broad-spectrum antibiotics administered throughout the study period, and also to the microaerobic conditions in the preterm intestine, which favors facultative anaerobes compared to strict anaerobes such as bifidobacteria (Arboleya et al., 2012). Similarly, delivery mode, which is a determinant of microbial colonization in term infants (Dominguez-Bello et al., 2010), had a minor effect in preterm infants, and it has been suggested that the NICU environment plays more of a role for shaping preterm gut microbiome (Brooks et al., 2017). Strains transmitted from the mother might also be unable to establish due to administration of antibiotics (Brooks et al., 2014). Inconsistencies between the results of different studies on preterm infant microbiota may result in part from differences in cohort inclusion criteria, such as range of patient gestational age or birth weight (Chernikova et al., 2018), as well as local differences in environmental microbial pools driven by factors such as seasonality or NICU hygiene practices (Taft et al., 2014).

In the initial microbial colonization of ELBWI, disturbances may also dramatically influence microbiome composition and make interpreting microbiome dynamics more challenging. In fact, the intermediate disturbance hypothesis posits that the magnitude and frequency of ecosystem disturbances can impact biological diversity (Hall et al., 2012), species dispersal, and colonization efficiency (Castorani and Baskett, 2020). In order to better understand initial assembly and succession processes in the face of these chaotic dynamics, we applied a probabilistic approach and analyzed state transitions through Markov chain modeling on our time-course data. Markov processes are widely used in many fields of science, from thermodynamics to phylogenetic inference and genome evolution (Erez et al., 2008; Kaehler et al., 2015; Sampid et al., 2018; Dhar et al., 2020; He et al., 2020), but have rarely been applied in microbial ecology (DiGiulio et al., 2015) and have not yet been used to evaluate longitudinal transitions in microbiome composition in an individual. Despite the large influence of stochastic processes, this analysis revealed distinctive microbiome dynamics, as well as community stability, for each body site (Figure 4).

## Conclusion

Moving forward, larger clinical studies are needed to establish the extent to which mono-dominated communities are associated with adverse outcomes and additional research is necessary to determine the mechanisms underlying this association. As sequencing technology now enables profiling of microbiomes within a few hours (Leggett et al., 2020), routine monitoring of neonate microbiomes coupled with time-integrated analysis of community diversity, structure and resilience, may prove to be a valuable complement to current diagnostic measurements. In summary, we have identified ecological factors determining the initial microbiome composition of oral cavity, gut, and skin samples of ELBWI and proposed a methodological framework for the analysis of microbiome dynamics based on Markov chain modeling. This framework has the potential to complement and refine existing clinical practices aimed at minimizing adverse outcomes in premature neonates.

## Data Availability Statement

The datasets presented in this study can be found in online repositories. The names of the repository/repositories and accession number(s) can be found below: https://www.ncbi.nlm.nih.gov/genbank/, PRJNA688751.

## Ethics Statement

The studies involving human participants were reviewed and approved by Ethics Committee of the Medical University of Vienna (No. 1175/2016). Written informed consent to participate in this study was provided by the participants’ legal guardian/next of kin.

## Author Contributions

LW and DB designed the study. CZ and LW acquired and processed samples and patient data. CZ, DS, FB, CH, AB, LW, and DB analyzed and interpreted data. CZ and DB drafted the manuscript, with input from all other authors. All authors have approved the submitted version and have agreed both to be personally accountable for their contributions and to ensure that questions related to the accuracy or integrity of any part of the work, even ones in which the author was not personally involved, are appropriately investigated, resolved, and the resolution documented in the literature.

## Conflict of Interest

The authors declare that the research was conducted in the absence of any commercial or financial relationships that could be construed as a potential conflict of interest.

## Publisher’s Note

All claims expressed in this article are solely those of the authors and do not necessarily represent those of their affiliated organizations, or those of the publisher, the editors and the reviewers. Any product that may be evaluated in this article, or claim that may be made by its manufacturer, is not guaranteed or endorsed by the publisher.
