# Supplementary material for: Ecological Processes Shaping Microbiomes of Extremely Low Birthweight Infants
Source: Front Microbiol. 2022 Feb 28;13:812136. doi: 10.3389/fmicb.2022.812136 (PMC8919028; doi:10.3389/fmicb.2022.812136)
Supplement: Supplementary file 1 [file Data_Sheet_1.docx]

**Supplementary Figures**


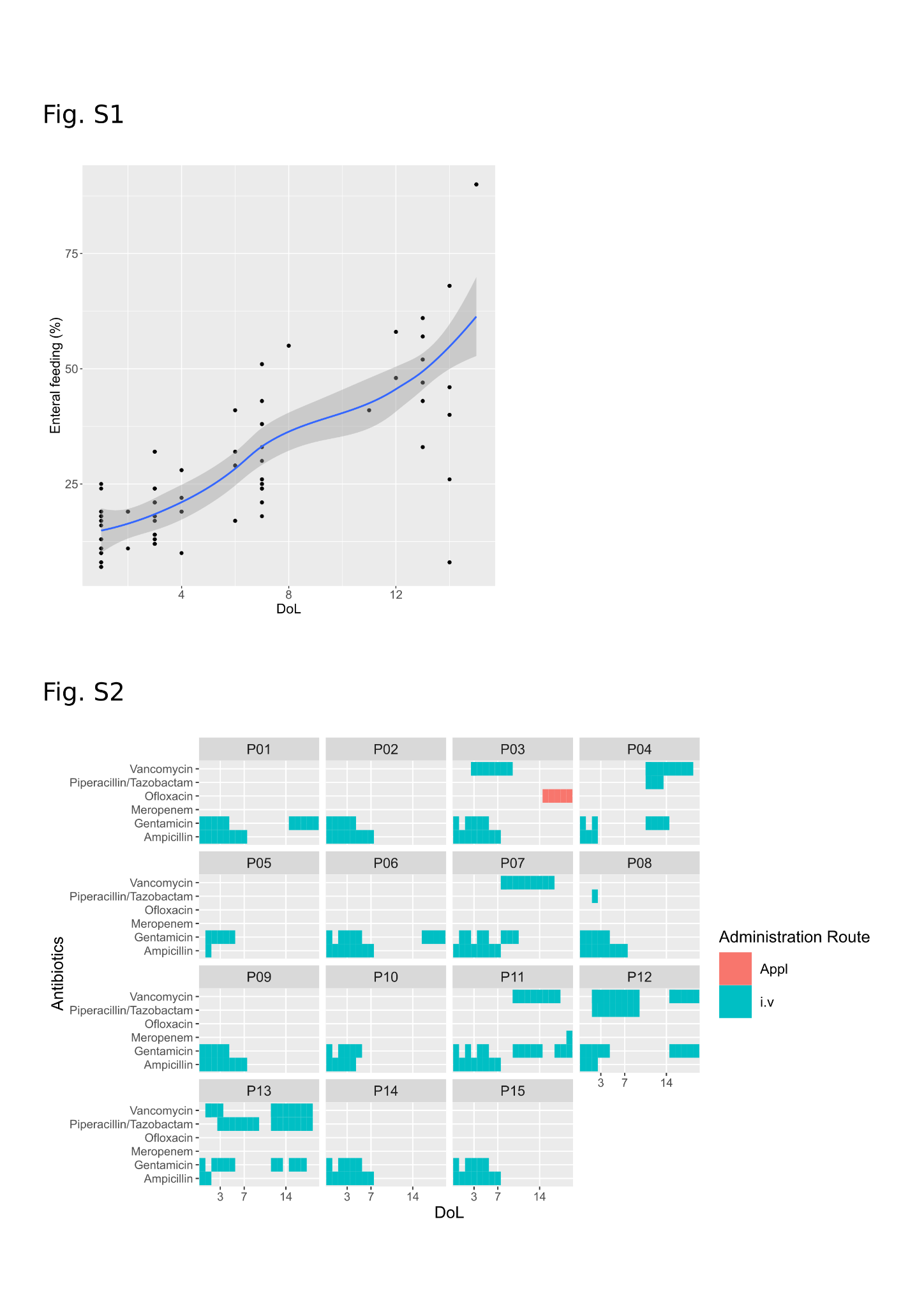


**Fig. S1. Percentage of enteral feeding during the study period.**

**
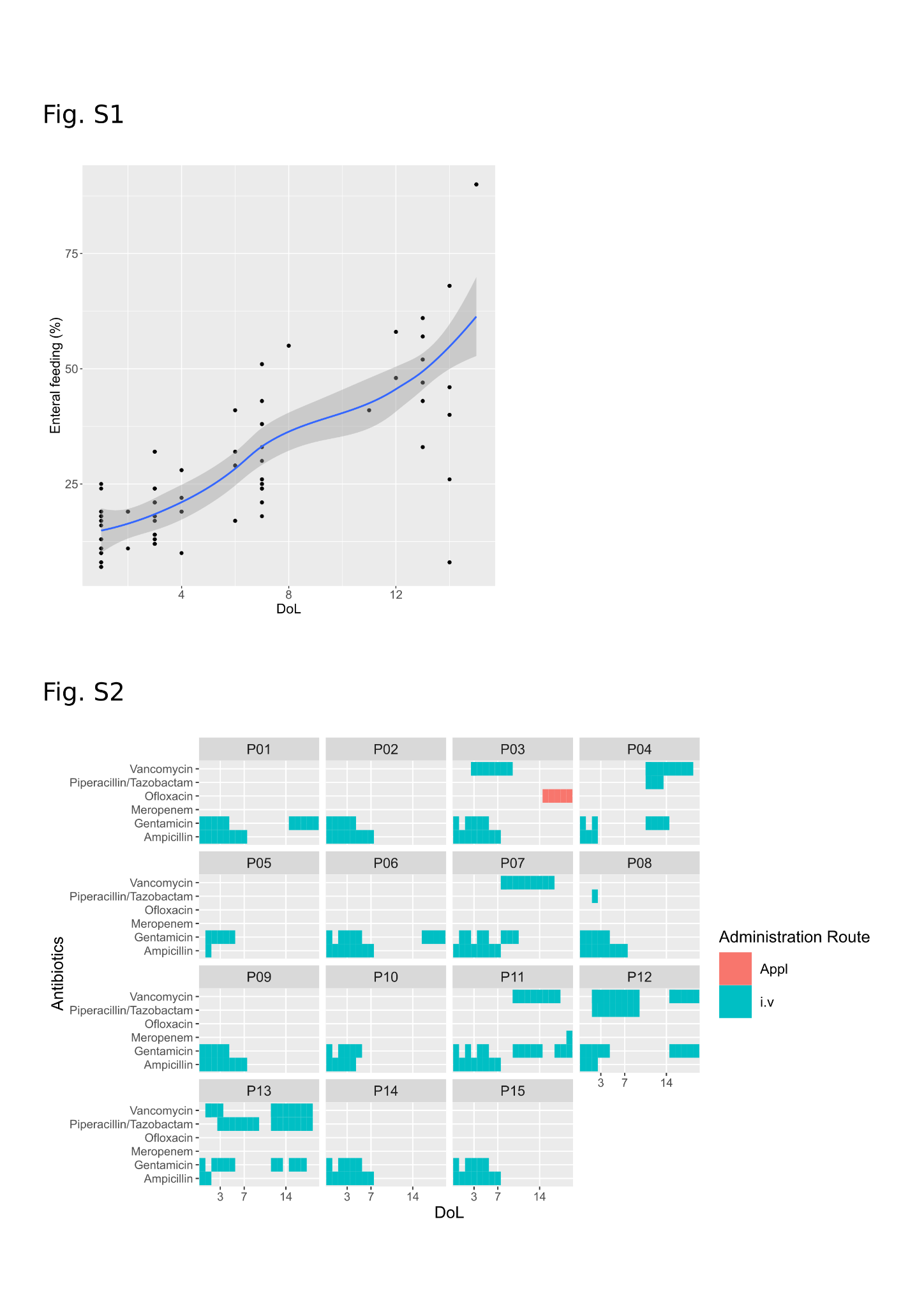
**

**Fig. S2. Antibiotics administration scheme for the study cohort.** I.v. = intravenous administration, Appl = external application.


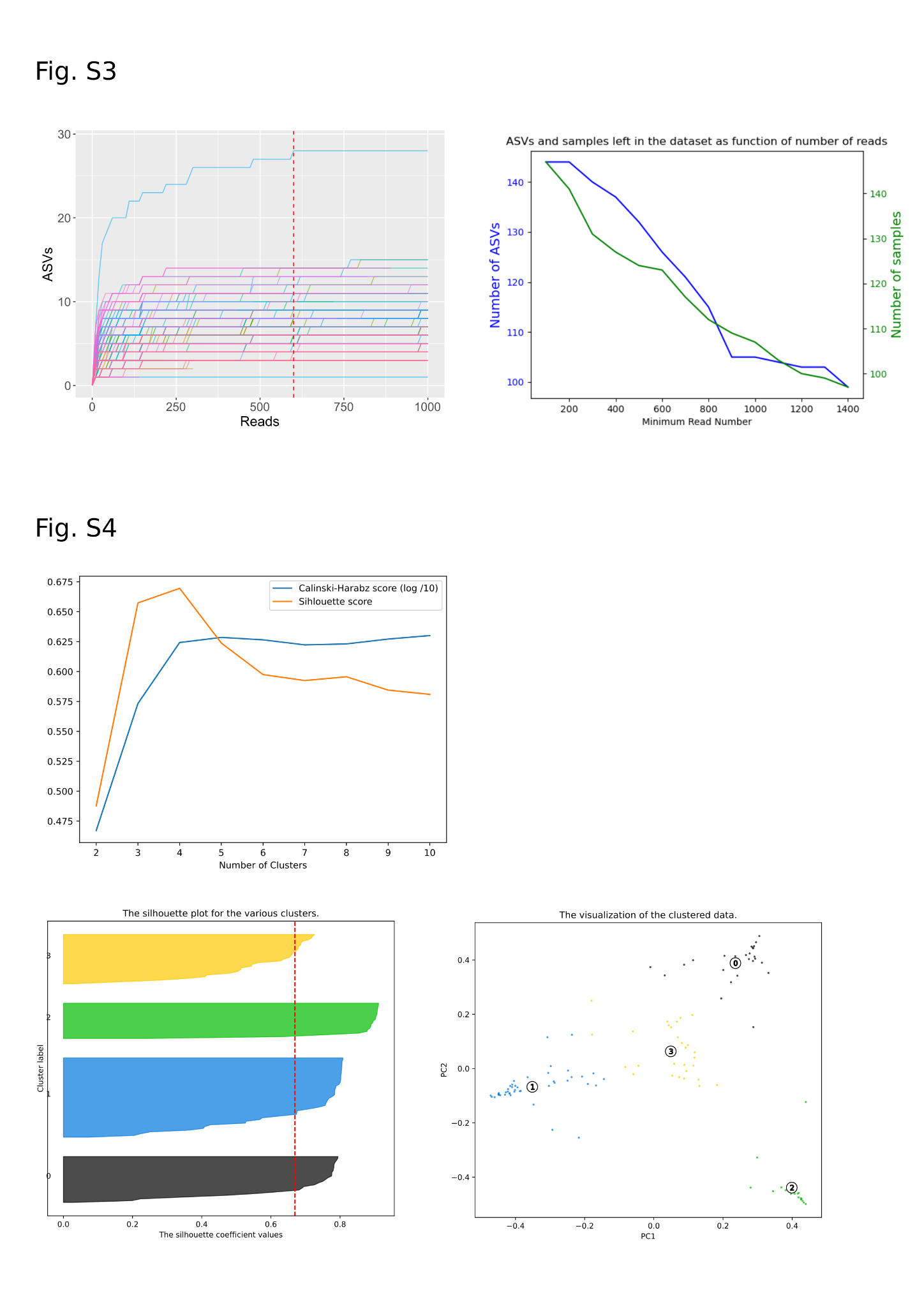


**Fig. S3. Criteria for selecting the sequence depth cutoff.** Rarefaction curves for each sample (left). Diagram of ASV and sample number included in the analysis for a range of sequence depths (right). Good's coverage min = 0.995, max = 1.0, CI_95_=[0.9986984 0.99918778]


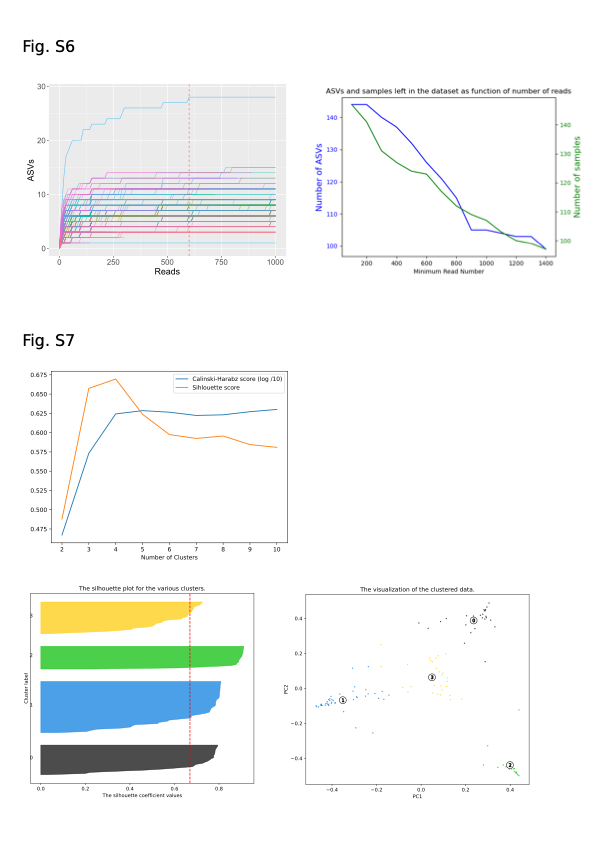


**Fig. S4. Clustering evaluation.** Silhouette and Calinski-Harabasz score for a range of clusters (top). Silhouette coefficient values for each cluster when four clusters are preselected for K-Means clustering (bottom).


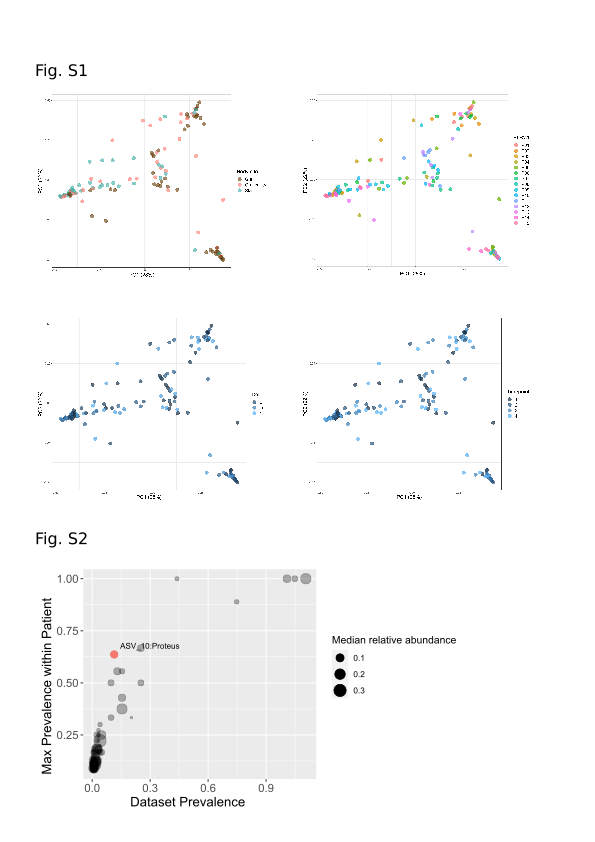


**Fig. S5. Most ASVs appear sporadically in the dataset.** ASV prevalence in the dataset calculated as a fraction of samples an ASV is detected (x-axis), compared to maximum ASV prevalence within one subject (y-axis). Size of circles shows ASV median relative abundance (including only samples that the ASV is detected). In red, an example of a *Proteus* ASV that is mainly detected in one subject (median relative abundance > 5%, dataset prevalence < 0.1 max subject prevalence > 0.6).

**
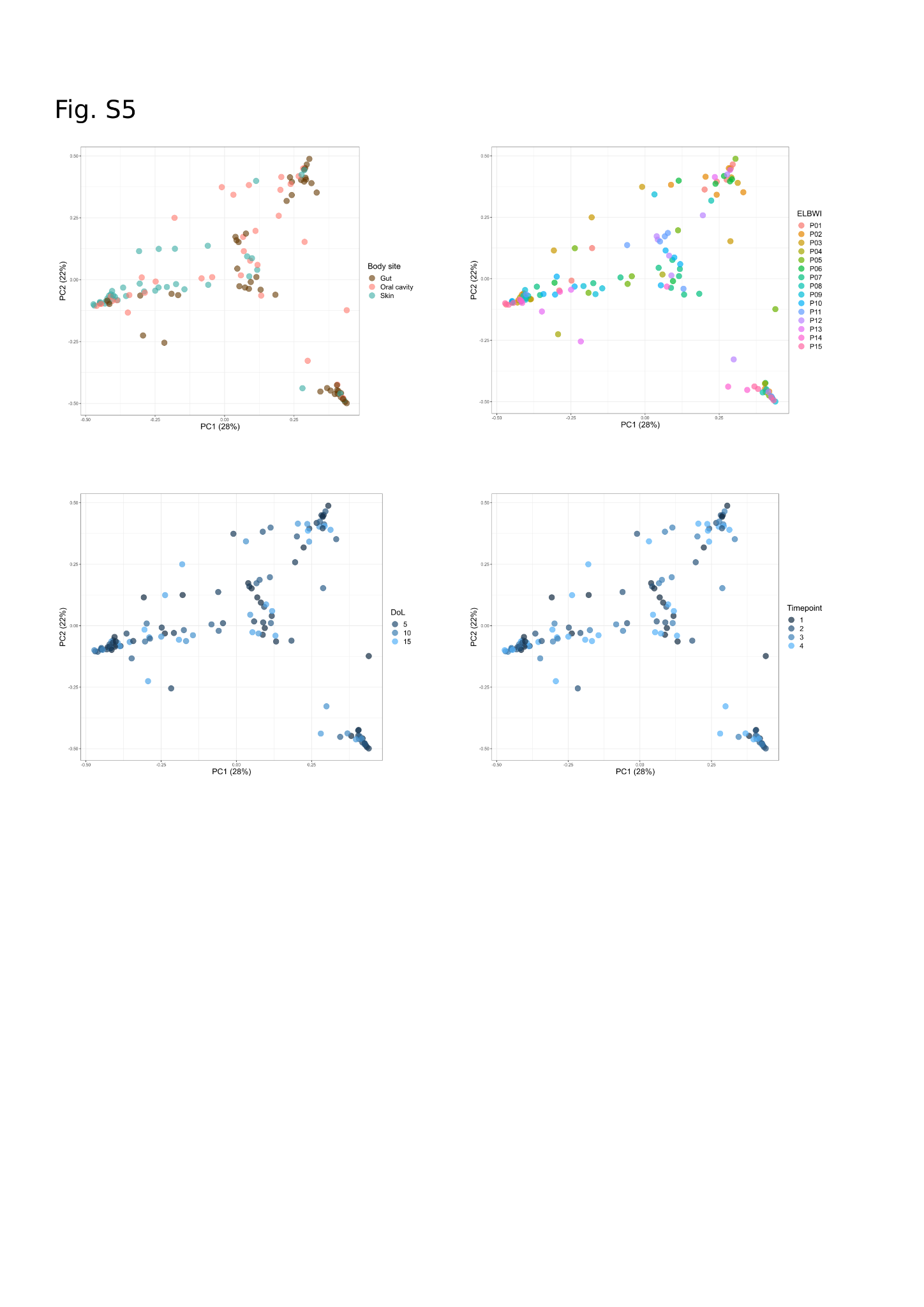
**

**Fig. S6. Sample do not cluster by body habitat, infant or days of life.** PCoA on Bray-Curtis dissimilarities colored for body site, Infant, Days of Life and time point of sampling, from left to right and top to bottom).


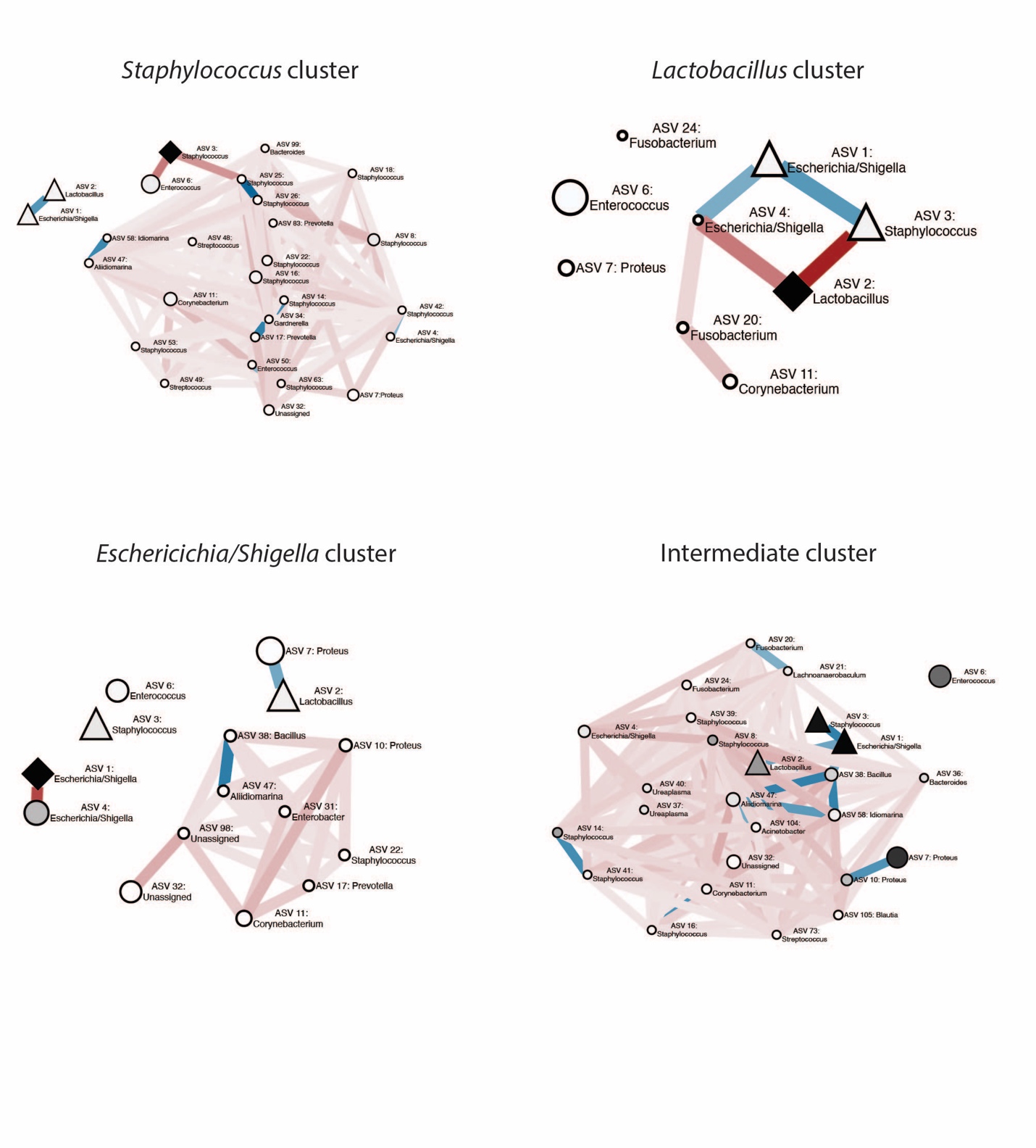


**Fig. S7. Co-occurrence networks for each community structure.** Edge colors indicate correlation direction (negative - red, positive - blue), edge color intensity indicates correlation strength, node size indicates species prevalence, node color indicates mean species abundance (low – white, black - high). Triangular nodes are mono-dominant species in any cluster. A diamond shape indicates the species that is mono-dominant in this specific cluster (if applicable).
